# Distinct Phenotypes of Inflammation Associated Macrophages and Microglia in the Prefrontal Cortex Schizophrenia Compared to Controls

**DOI:** 10.3389/fnins.2022.858989

**Published:** 2022-06-30

**Authors:** Yunting Zhu, Maree J. Webster, Caitlin E. Murphy, Frank A. Middleton, Paul T. Massa, Chunyu Liu, Rujia Dai, Cyndi Shannon Weickert

**Affiliations:** ^1^Department of Neuroscience, SUNY Upstate Medical University, Syracuse, NY, United States; ^2^Stanley Medical Research Institute, Rockville, MD, United States; ^3^Schizophrenia Research Laboratory, Neuroscience Research Australia, Sydney, NSW, Australia; ^4^Department of Neurology and Microbiology and Immunology, SUNY Upstate Medical University, Syracuse, NY, United States; ^5^Department of Psychiatry, SUNY Upstate Medical University, Syracuse, NY, United States; ^6^School of Psychiatry, Faculty of Medicine, University of New South Wales, Sydney, NSW, Australia

**Keywords:** neuroinflammation, microglia, macrophage, schizophrenia, dorsolateral prefrontal cortex

## Abstract

Approximately 40% of people with schizophrenia are classified as having “high inflammation.” This subgroup has worse neuropathology than patients with “low inflammation.” Thus, one would expect the resident microglia and possibly monocyte-derived macrophages infiltrating from the periphery to be “activated” in those with schizophrenia with elevated neuroinflammation. To test whether microglia and/or macrophages are associated with increased inflammatory signaling in schizophrenia, we measured microglia- and macrophage-associated transcripts in the postmortem dorsolateral prefrontal cortex of 69 controls and 72 people with schizophrenia. Both groups were stratified by neuroinflammatory status based on cortical mRNA levels of cytokines and SERPINA3. We found microglial mRNAs levels were either unchanged (IBA1 and Hexb, *p* > 0.20) or decreased (CD11c, <62% *p* < 0.001) in high inflammation schizophrenia compared to controls. Conversely, macrophage CD163 mRNA levels were increased in patients, substantially so in the high inflammation schizophrenia subgroup compared to low inflammation subgroup (>250%, *p* < 0.0001). In contrast, high inflammation controls did not have elevated CD163 mRNA compared to low inflammation controls (*p* > 0.05). The pro-inflammatory macrophage marker (CD64 mRNA) was elevated (>160%, all *p* < 0.05) and more related to CD163 mRNA in the high inflammation schizophrenia subgroup compared to high inflammation controls, while anti-inflammatory macrophage and cytokine markers (CD206 and IL-10 mRNAs) were either unchanged or decreased in schizophrenia. Finally, macrophage recruitment chemokine CCL2 mRNA was increased in schizophrenia (>200%, *p* < 0.0001) and CCL2 mRNA levels positively correlated with CD163 mRNA (*r* = 0.46, *p* < 0.0001). Collectively, our findings support the co-existence of quiescent microglia and increased pro-inflammatory macrophages in the cortex of people with schizophrenia.

## Introduction

Mounting molecular evidence indicates that dysregulation of the immune system may play a significant role in the pathogenesis of schizophrenia (van Kesteren et al., 2017). Variation in immune-related genes has been linked to an increased risk for developing schizophrenia, including genes within the major histocompatibility molecule (MHC) locus (Schizophrenia Psychiatric Genome-Wide Association Study [GWAS] Consortium, 2011) and genes encoding specific inflammatory cytokines (Bocchio Chiavetto et al., 2002; Schwarz et al., 2006, 2014). Further, we and others have found elevated mRNA levels of pro-inflammatory cytokines and immune regulators in postmortem cortical and subcortical brain tissue from people with schizophrenia. These changes included increases in interleukin (IL)-1β, IL-6, IL-8 (Fillman et al., 2013; Volk et al., 2015; Pandey et al., 2018), Serpin Family A Member 3 (SERPINA3), interferon-induced transmembrane proteins 2 and 3 (IFITM2 and IFITM3) and nuclear factor kappa-light-chain-enhancer of activated B cells (NF-κB) (Arion et al., 2007; Saetre et al., 2007; Fillman et al., 2013, 2014; Volk et al., 2015, 2019; Zhang et al., 2016; Volk, 2017; Cai et al., 2018; Purves-Tyson et al., 2019; Murphy et al., 2020b; Weissleder et al., 2021). Increases in mRNA and protein levels of cytokines in the blood of living patients are also consistently reported, suggesting some degree of both peripheral and central (brain) immune activation in people with schizophrenia (Kowalski et al., 2001; Theodoropoulou et al., 2001; Potvin et al., 2008; Miller et al., 2011; Perkins et al., 2015; Boerrigter et al., 2017; Hoseth et al., 2017; Di Biase et al., 2021).

Despite evidence of neuroinflammation in at least 40% of patients (Fillman et al., 2013, 2014; Boerrigter et al., 2017; Purves-Tyson et al., 2019; Lizano et al., 2020; Weissleder et al., 2021), which we defined as the “high inflammation” schizophrenia subgroup, the brain-resident immune cells—microglia—do not appear to be activated in the prefrontal cortex in schizophrenia in a way that is typical of other neuroinflammatory conditions. While earlier studies produced conflicting evidence regarding microglial activation in schizophrenia (Bayer et al., 1999; Falke et al., 2000; Wierzba-Bobrowicz et al., 2005; Foster et al., 2006; Nakatani et al., 2006; Steiner et al., 2006, 2008; Saetre et al., 2007; Connor et al., 2009; Kano et al., 2011; Busse et al., 2012; Fillman et al., 2013; Sinkus et al., 2013; Gos et al., 2014; Durrenberger et al., 2015) cohorts have shown that microglial networks appear downregulated in people with schizophrenia, which co-occurs with an overall up-regulation of inflammatory pathways (Gandal et al., 2018b; Toker et al., 2018). This raises the possibility that cortical microglia are suppressed in the microenvironment of people with schizophrenia and cortical inflammation. However, normally microglia are more activated during acute inflammatory conditions (Streit et al., 2004; E Hirbec et al., 2017; Sousa et al., 2018). The notion of putative microglial suppression in schizophrenia is further bolstered by studies showing higher densities of dystrophic and degenerative microglia with damaged processes (Wierzba-Bobrowicz et al., 2005; Clare et al., 2021) and increased markers of cellular senescence within microglia, including decreased volume fraction of mitochondria and increased area of lipofuscin granules (Uranova et al., 2021). Interestingly, though, other evidence supports over-activity of microglia in schizophrenia that may differ between cortical and subcortical regions (prefrontal cortex vs. midbrain), and between gray and white matter (Purves-Tyson et al., 2020; Gober et al., 2021), and which may vary depending on stage of illness (first episode vs. chronic) (Bloomfield et al., 2016). These inconsistent results might also be contributed from the proportion of people with elevated inflammation in the studies. High inflammation subgroups in schizophrenia and controls may have different phenotypes of microglia, and combining high and low inflammation subgroups could mask the potential differences between these subgroups within diagnostic categories.

The fact that the state of microglia may be highly variable and possibly even suppressed concomitant with elevations in cytokines, suggests that other cell types may contribute to neuroinflammation in schizophrenia, potentially myeloid cells. One type of myeloid cells in the brain, macrophages, are poised to make major contributions to inflammatory transcript levels as they synthesize high levels of many inflammatory factors (Varvel et al., 2016; Park et al., 2020). Indeed, we recently found elevated mRNA and protein levels of the macrophage marker CD163 in the brains of people with schizophrenia, and this increase was associated with changes in endothelial gene expression (Cai et al., 2018; Purves-Tyson et al., 2020; Weissleder et al., 2021). Further, circulating macrophages can enter the brain under conditions of neuroinflammation (Minogue, 2017), and we found parenchymal and perivascular CD163+ macrophages were increased in density in the high inflammation subgroup of schizophrenia patients (Purves-Tyson et al., 2020; Weissleder et al., 2021). The changes in endothelial genes and increased density of CD163+ macrophage may suggest that more macrophages are infiltrating the brain specifically in high inflammation schizophrenia subgroup, but it is not known if this would also occur in normal controls defined as having high inflammation.

There is lack of clarity concerning which molecular markers can be best used to distinguish microglia from macrophages. For example, the commonly used microglial markers (HLA, CD68, and IBA1) are also expressed by perivascular macrophages (Faraco et al., 2017; Swanson et al., 2020). The inconsistency of microglial marker changes in schizophrenia could therefore be due to the use of single markers to measure microglia activity/density. Another commonly used “microglial marker,” TSPO, can be expressed by macrophages and astrocytes (De Picker and Morrens, 2020; Notter et al., 2020). Therefore, we measured multiple microglial markers including TSPO and macrophage marker CD163 mRNA levels to compare the molecular phenotypes of microglia and macrophages between schizophrenia and normal controls as well as between high and low inflammation subgroups. We also measured transcript levels of the reactive astrocyte marker GFAP since cortical astrogliosis may exist in a subset of people with schizophrenia (Webster et al., 2005; Feresten et al., 2013; Catts et al., 2014; Toker et al., 2018; Murphy et al., 2020a) and reactive astrocytes reciprocally influence microglia polarization states (Jha et al., 2019; Corsi-Zuelli and Deakin, 2021).

Macrophages and microglia exist along a spectrum of phenotypes, with “M1-like” pro-inflammatory and “M2-like” anti-inflammatory phenotypes representing the extremes of this spectrum (Xue et al., 2014), and it is not currently known which macrophage phenotype predominates in the cortex of people with schizophrenia. Macrophages may dampen neuroinflammation emanating from damaged tissue or, conversely, may worsen or even cause neuroinflammation in the brain. However, macrophages in the normal healthy brain may exist as a different phenotype, maintaining a balanced inflammation and immune response during inflammation. Though CD163 is often used as a macrophage marker, it cannot reliably differentiate between macrophage phenotypes (Roberts et al., 2004; Kim et al., 2006; Holfelder et al., 2011; Zhang et al., 2011, 2012; Lisi et al., 2017) and is thus not useful in distinguishing macrophage type changes in high inflammation schizophrenia. Typical “M1-like” macrophages express CD64 mRNA, whereas the “M2-like” macrophages express CD206 mRNA and the anti-inflammatory cytokine IL-10 mRNA. The ratio of CD64 mRNA and CD206 mRNA could indicate whether pro- or anti-inflammatory macrophages are more likely to be found in high inflammation schizophrenia subgroup. One type of regulatory macrophage (M2), M2b, can secrete some cytokines known to be elevated in schizophrenia including IL-1, IL-6, and TNF-a (Fillman et al., 2013; Volk et al., 2015; Zhang et al., 2016). Therefore, we measured the following pro- and anti- inflammatory macrophage marker mRNAs : CD64, CD206, IL-10, and CD86 (M2b marker) in the dorsolateral prefrontal cortex of people with schizophrenia and unaffected controls. We hypothesized that the macrophage recruitment chemokine (CCL2/MCP-1) (Semple et al., 2010; Gschwandtner et al., 2019), and the macrophage-derived chemokine IL-8 would be elevated in the high inflammation schizophrenia cortex (Huber et al., 1991; Bell et al., 1996), and we performed regression analysis to predict which macrophage types are more related to CCL2.

Here, we measured multiple microglia, astrocyte and macrophage marker mRNA levels in the prefrontal cortex in people with schizophrenia and controls (stratified into low and high inflammation subgroups in both diagnostic groups). This allowed us to ask what, if any, changes may be specific to high inflammation schizophrenia as compared to high inflammation controls. We anticipated that macrophage and astrocyte markers would be elevated in high inflammation schizophrenia rather than microglia markers compared to high inflammation controls. We further predicted that one of the macrophage markers, CD163 mRNA, would correlate with the “M1-like” marker CD64, and that these increases in pro-inflammatory macrophage markers may be specific to high inflammation schizophrenia subgroup. Given that the macrophage chemoattractant CCL2 promotes pro-inflammatory “M1-like” polarization in human macrophages (Sierra-Filardi et al., 2014), we also expected that CCL2 mRNA to be increased in high inflammation schizophrenia and to be positively related to proinflammatory macrophage markers and negatively related to anti-inflammatory macrophage markers (Yang et al., 2011; Gschwandtner et al., 2019). Furthermore, we compared our mRNA levels between diagnostic groups with the larger cohort RNA sequencing data from PsychENCODE Consortium to determine if we could determine the diagnostic specificity of our main diagnostic effects.

## Materials and Methods

### Human Post-mortem Dorsolateral Prefrontal Cortex Tissue Collection and Demographics

Human post-mortem brain DLPFC tissue (Brodmann Area 46) was obtained from the New South Wales Brain Tissue Resource Centre (TRC) and Stanley Medical Research Institute (SMRI). The final mRNA cohort of this study included 72 schizophrenia cases and 69 normal controls. The detailed demographics are shown in Table 1. The age, sex, brain hemisphere, postmortem interval (PMI), and RNA integrity number (RIN) were matched between diagnostic groups. The mean pH was slightly lower in the schizophrenia group (*p* = 0.07). Our study was approved by the Human Research Ethics Committee at University of NSW (#HREC: HC12435; HC17826).

**TABLE 1:** Comparison of demographic variables between diagnostic groups.

Demographics	Control	Schizophrenia	t/U/χ ^2^ (df)	*P-value*
	(*n* = 69)	(*n* = 72)		
Age in years ± s.d.	48.01 ± 12.17	47.07 ± 12.45	*t*(139) = 0.46	0.65
Sex	16F:53M	22F:50M	χ^2^(1) = 0.97	0.32
Hemisphere	40R:29L	35R:37L	χ^2^(1) = 1.24	0.27
Brain pH ± s.d.	6.63 ± 0.28	6.55 ± 0.28	*t*(139) = 1.85	0.067
PMI (hours) ± s.d.	27.15 ± 12.31	29.93 ± 14.63	*U* = 2225.00	0.29
RIN ± s.d.	7.73 ± 0.79	7.85 ± 0.83	*t*(139) = 0.92	0.36
Age (years) at onset ± s.d.	−	22.53 ± 6.15	−	−
Duration of illness (years) ± s.d.	−	24.54 ± 12.50	−	−
Chlorpromazine mean equivalent daily dose (mg) ± s.d.	−	616.03 ± 502.71	−	−

*PMI, postmortem interval; RIN, RNA integrity number.*

All cases in the cohort were previously categorized into low or high inflammation subgroups using a two-step recursive clustering analysis based on mRNA expression of SERPINA3, IL-1β, IL-6, and IL-8 (Fillman et al., 2013, 2014). The combined cohort used in this study consisted of 57 low inflammation controls (CON-low), 12 high inflammation controls (CON-high), 42 low inflammation schizophrenia (SCZ-low), and 30 high inflammation schizophrenia (SCZ-high). Demographic comparisons between inflammation subgroups are shown in Table 2. Age, sex, PMI, and RIN were matched across all the inflammation subgroups. However, brain pH was significantly lower in both high inflammation subgroups (*p* < 0.0001), consistent with the acidotic effects of inflammation in tissue (Erra Diaz et al., 2018).

**TABLE 2:** Comparison of demographic variables between inflammation subgroups.

Demographics	Control low (*n* = 57)	Control high (*n* = 12)	Schizophrenia low (*n* = 42)	Schizophrenia high (*n* = 30)	F/χ ^2^/U/t	*P-value*
Age in years ± s.d.	48.53 ± 11.49	45.58 ± 15.33	46.38 ± 13.57	48.03 ± 10.84	*F*_(3,137)_ = 0.36	0.78
Sex	12F:45M	4F:8M	15F:27M	7F:23M	χ^2^(3) = 3.09	0.38
Hemisphere	33R:24L	7R:5L	18R:24L	17R:13L	χ^2^(3) = 2.58	0.46
Brain pH ± s.d.	6.69 ± 0.25	6.37 ± 0.31	6.67 ± 0.22	6.37 ± 0.27	*F*_(3,137)_ = 15.17	1.41E-8
PMI (hours) ± s.d.	27.45 ± 12.30	25.75 ± 12.76	31.07 ± 11.68	28.22 ± 18.06	χ^2^(3) = 3.56	0.31
RIN ± s.d.	7.71 ± 0.75	7.86 ± 0.98	7.92 ± 0.86	7.77 ± 0.78	*F*_(3,137)_ = 0.58	0.63
Age (years) at onset ± s.d.	−	−	23.45 ± 6.93	21.23 ± 4.65	*U* = 515.50	0.19
Duration of illness (years) ± s.d.	−	−	22.93 ± 13.31	26.80 ± 11.09	*t*(70) = −1.30	0.20
Chlorpromazine mean equivalent daily dose (mg) ± s.d.	−	−	515.34 ± 428.24	757.00 ± 569.38	*U* = 463.5	0.057

*PMI, postmortem interval; RIN, RNA integrity number.*

### RNA Extraction, Complementary DNA Synthesis, and Quantitative PCR

Total RNA was extracted from fresh frozen DLPFC postmortem brain tissue using the TRIzol (Invitrogen, Carlsbad, CA, United States) extraction method. RNA concentration was determined by Agilent Technologies 2100 Bioanalyzer. Complementary DNA (cDNA) synthesis was performed from 1 μg total RNA per case using SuperScript III First-Strand Synthesis kit (Life Technologies, Scoresby, VIC, Australia). The mRNA expression of microglia, astrocyte, macrophage, cytokine and chemokine transcripts were measured by reverse transcriptase-quantitative PCR using Fluidigm BioMark™ HD system (South San Francisco, CA, United States) at the Ramaciotti Centre for Genomics (Kensington, NSW, Australia) using pre-designed Taqman Gene Expression Assays that included: (1) Ionized calcium binding adaptor molecule 1 (IBA1) (Hs00741549_g1), (2) integrin alpha X (ITGAX/CD11c) (Hs00174217_m1), (3) hexosaminidase subunit beta (Hexb) (Hs01077594_m1), (4) cluster of differentiation 68 (CD68) (Hs00154355_m1), (5) translocator protein (18kD) (TSPO) (Hs00559362_m1), (6) astrocyte marker glial fibrillary acidic protein (GFAP) (Hs00909233_m1), (7) CD163 (Hs00174705_m1), (8) CD64 (Hs00174081_m1), (9) CD206 (Hs00267207_m1), (10) CD86 (Hs01567026_m1); (11) IL-10 (Hs00961622_m1), (12) CCL2 (Hs00234140_m1), and (13) IL-8 (Hs00174103_m1). No template controls and no reverse transcriptase controls were included in the assays to test for reagent contamination and for genomic DNA amplification, respectively. Relative quantity of mRNA expression of each gene was calculated using 2-ΔΔCt method. First, the ΔCt of each sample was calculated by subtracting the geometric means of three housekeeper transcripts [(1) glyceraldehyde 3-phosphate dehydrogenase (Hs99999905_m1); (2) TATA-binding protein (Hs00427620_m1); (3) ubiquitin C (Hs00824723_m1)] from Ct value of the target gene (ΔCt = Ct value-geometric mean of housekeeper transcript Ct value). Then, ΔΔCt was calculated by subtracting the average ΔCt of the control from each sample’s ΔCt (ΔΔCt = ΔCt − control average ΔCt). The final relative mRNA expression for all the samples were calculated by the formula 2^–Δ^ ^Δ^ *^Ct^*.

### Statistical Analysis

Dependent variables were tested for normality using Kolmogorov–Smirnov test. Independent samples *t*-tests (for normal data), Mann-Whitney *U* tests (for non-normal data), and Chi-square tests were used to detect differences in demographic characteristics between diagnostic groups. Analysis of variance (ANOVA) and Chi-square tests were used to detect differences in demographic characteristics among inflammation subgroups. Independent samples t-tests and Mann-Whitney *U* tests were used to compare the age at onset, duration of illness and chlorpromazine (CPZ) equivalent daily dose between schizophrenia low and high inflammation subgroups.

For mRNAs of interest, each transcript (expressed as 2-ΔΔCt) were tested for normality using Shapiro–Wilk test and Kolmogorov–Smirnov test. Normal Q-Q plots were also used to determine whether the data was normally distributed. If the distribution of the data was not normal, log transformation was conducted. Homogeneity of variance was tested using Levene’s test. Outliers were removed if the normalized expression value fell beyond two standard deviations of the means for the group/subgroup. Pearson’s correlations were performed between demographic variables (age, PMI, brain pH, and RIN) and each mRNA of interest (either normalized data or log transformed data) to identify potential covariates for subsequent analyses. If a normalized transcript was correlated with any demographic variable [excluding brain pH (Hagihara et al., 2018)], a two-way analysis of covariance (ANCOVA) was performed to examine the effect of diagnosis, inflammation and the interaction of diagnosis and inflammation on the gene expression [all correlations (Pearson’s r) and *p*-values shown in Supplementary Table 3]. The homogeneity of regression slopes assumption was confirmed by analyzing the interaction between the covariate and grouping variables (*p* > 0.05). If no covariates were identified, data were analyzed using two-way ANOVA. If data was extremely skewed based on normality testing and normal Q-Q plot (CCL2 mRNA), non-parametric factorial ANOVA (aligned rank transformation analysis of variance) was performed using align-and-rank transformed data (Wobbrock et al., 2011). Pairwise comparison was conducted by a Mann–Whitney *U* test.

If interaction effect (diagnosis or inflammation) reached statistical significance (*p* < 0.05), pairwise *post hoc* comparisons (main effects) were performed and, Fisher’s least significant difference (LSD, for interactions) *post hoc* tests were performed. Since our priori hypothesis was that there may be changes specific to high inflammation schizophrenia as compared to high inflammation controls, we performed pairwise comparison between these two groups even if the interaction effect did not reach statistical significance.

For correlation analyses of microglia, macrophage and astrocyte markers, Pearson’s correlation was performed between levels for each of the following transcripts: Hexb, CD11c, IBA1, CD68, CD163, CD64, CD86, CD206, GFAP, and TSPO. Since the correlation analyses were exploratory, all samples were included in the analysis. We also performed correlation analysis between our microglial marker IBA1 mRNA and data from the Stanley Neuropathology Consortium Integrative Database [Array Collection deposited by Clare Beasley IBA1+ cells in the frontal cortex^1^ (Kim and Webster, 2010)]. The data was based on IBA1+ cell density in all six layers in the cortex gray matter. We calculated the average IBA1+ cell density per total DLPFC by accounting for the differential contribution of each layer in human cortex to the total surface area of the cortex (He et al., 2017) (average cell density = Layer 1 density [L1]*12.3% + L2*6.13% + L3*35.93% + L4*6.41% + L5*14.59% + L6*24.96%) (*n* = 60). Pearson’s correlation analysis was performed on IBA1 mRNA levels (only the cases with IBA mRNA who also had IBA1+ cell density in frontal cortex gray matter were included, *n* = 55).

To determine the relationships between cell markers and inflammatory markers, we performed stepwise multiple linear regression analysis, where the criteria for a predictor to enter into the analysis is based on partial *F* test *p*-value ≤ 0.05, and to be removed from the analysis is *p* ≥ 0.10. All the cases were included in this analysis. The final model was determined when no more predictors could be entered or removed from the model. First, to determine whether TSPO mRNA was more related to microglia (IBA1), astrocyte (GFAP), or macrophage (CD163) marker levels, the regression model included TSPO as the response variable, and IBA1, GFAP, and CD163 levels as predictors. Second, to determine whether CD163 mRNA was more likely to correlate with pro-inflammatory, anti-inflammatory macrophage marker or microglia mRNAs, we performed stepwise linear regression analysis on CD163 as the response variable and CD64, CD206, and IBA1 levels as predictors. Thirdly, to test which macrophage type was more likely to associate with macrophage chemokine CCL2, multiple macrophage markers including CD163, CD64, CD68, CD206, and CD86 were used as predictors in the linear regression model with CCL2 levels as the response variable.

All statistical analyses and data visualization were performed using R (version 4.0.2) and Prism (v8, GraphPad, La Jolla, CA, United States).

### PsychENCODE Consortium RNA Sequencing Data Analysis

We used postmortem-brain DLPFC RNAseq data in PsychENCODE Consortium [PubMed ID: 30545856, doi: doi.org/10.7303/syn1208024] to test two things (1) if the diagnostic changes in inflammatory mRNAs we detected in schizophrenia could be reproduced and (2) if similar diagnostic changes could be detected in bipolar disorder and autism telencephalon. The samples are collected from the frontal cortex and temporal cortex. Low expressed genes with transcripts per million (TPM) < 0.1 in more than 25% samples were filtered and mitochondria genes were removed. Samples with standardized network connectivity Z scores < 2 were included–two samples were defined as outlier samples and removed. The samples with discordant sex information were also removed. In total, 25,774 genes and 2,160 samples were retained. The data included 1,232 healthy controls, 593 schizophrenia samples, 253 bipolar disorders samples, and 82 autism samples. We identified factors such as age, sex, batches, PMI, RIN, brain bank, brain region, and sequencing-related principal components as known covariates. Hidden covariates were identified by surrogate variable analysis (SVA). Count matrix were corrected for library size using Trimmed Mean of the M-values (TMM) normalization in edgeR and was log-transformed. Differentially expressed transcripts were calculated with the linear mixed-effects model using nlme packages in R. The unknown and hidden covariates were included in the model. The *p*-values were corrected by Benjamini–Hochberg method.

## Results

### Microglial mRNA Markers Are Unchanged or Reduced in High Inflammation Schizophrenia

**FIGURE 1:**
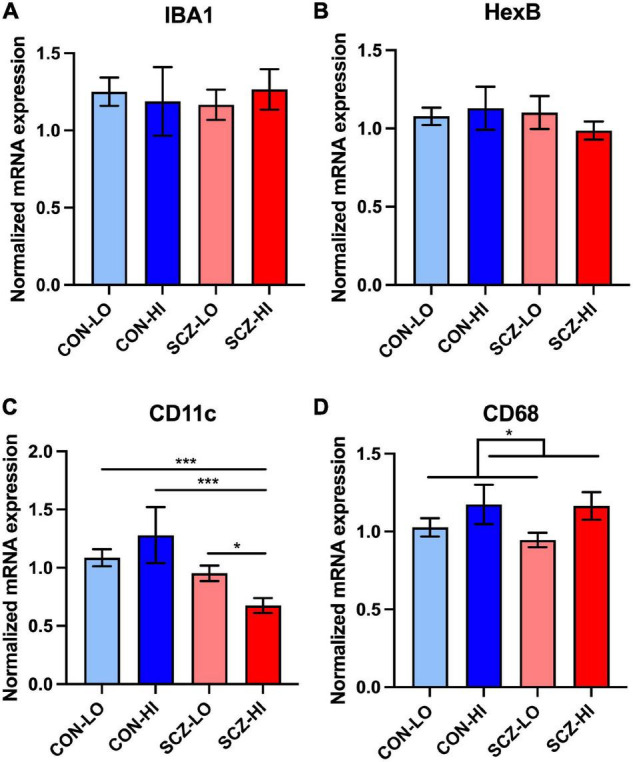
Microglia-related gene expression in DLPFC across inflammation subgroups. **(A,B)** There was no significant difference between diagnostic groups or inflammation groups in IBA1 and Hexb expression. **(C)** There was an interaction effect of diagnosis and inflammation in CD11c expression [*F*_(1,130)_ = 5.27, *p* = 0.023], that was also significantly decreased in schizophrenia high inflammation subgroup compared to all other inflammation subgroups. **(D)** CD68 expression was elevated in high inflammation group (*p* = 0.039) compared to low inflammation group. Error bars depict standard error of the mean. *p < 0.05, ***p < 0.001.

We measured gene expression of four microglial markers in the DLPFC: IBA1, Hexb, CD11c, and CD68. We found that neither diagnosis [IBA1: *F*_(1,128)_ = 1.04, *p* = 0.31; Hexb: *F*_(1,129)_ = 0.19, *p* = 0.67] or inflammatory status [IBA1: *F*_(1,128)_ = 1.27, *p* = 0.26; Hexb: *F*_(1,129)_ = 0.122, *p* = 0.73] had a significant main effect on IBA or Hexb mRNA levels (Figures 1A,B). We found no interaction effect (diagnosis × inflammation) for these mRNAs [IBA1: *F*_(1,128)_ = 5.13E-04, *p* = 0.98; Hexb: *F*_(1,129)_ = 0.477, *p* = 0.49]. There was no significant difference between SCZ-high and CON-high subgroups (IBA1: *p* = 0.59, Hexb: *p* = 0.35). We confirmed that our IBA1 mRNA levels were positively correlated with IBA1+ cell density defined morphologically as microglia reported previously [Array Collection deposited by Clare Beasley IBA1+ cells in the frontal cortex (see text foot note 1) (Kim and Webster, 2010)]. In the prefrontal cortex of the same cases, IBA1 mRNA correlated with total IBA1+ microglia density (*r* = 0.27, *p* = 0.043), but did not correlate with ramified or non-ramified IBA1+ microglia densities (*p* > 0.05).

In contrast to pan microglial markers, CD11c gene expression was significantly decreased in the schizophrenia group compared to controls overall [main effect of diagnosis: *F*_(1,130)_ = 12.95, *p* = 4.54E-04] (Figure 1C). We found no significant inflammation effect on CD11c mRNA levels [main effect of inflammation: *F*_(1,130)_ = 0.17, *p* = 0.62]. We also detected a diagnosis × inflammation interaction effect on CD11c expression [*F*_(1,130)_ = 5.27, *p* = 0.023]. When comparing inflammation subgroups, CD11c mRNA levels were decreased in the SCZ-high subgroup (SCZ-high) compared to all of the other three groups [CON-low (*p* < 0.001), CON-high (*p* < 0.001), and SCZ-low (*p* = 0.025) (Figure 1C)]. CD68 mRNA showed no significant change by diagnosis [*F*_(1,128)_ = 0.20, *p* = 0.66], but was significantly higher in high inflammation individuals compared to low inflammation individuals [main effect of inflammation: *F*_(1,128)_ = 4.34, *p* = 0.039] (Figure 1D). There was no diagnosis × inflammation interaction effect on CD68 expression [*F*_(1,128)_ = 0.017, *p* = 0.90]. There was no significant difference in CD68 expression between SCZ-high and CON-high subgroups (*p* = 0.86).

### Transcripts Expressed by Microglia, Astrocytes, and/or Macrophages Are Elevated in High Inflammation Schizophrenia Subgroup

**FIGURE 2:**
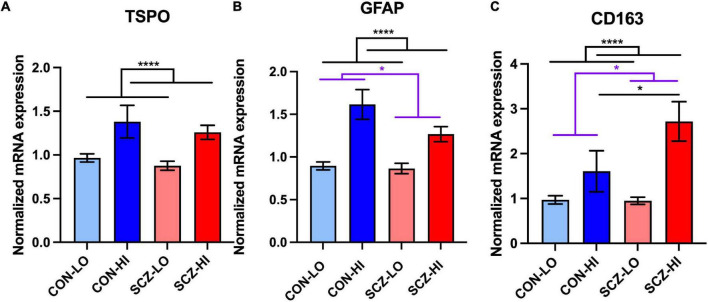
Microglia, astrocyte, and macrophage marker mRNA expression and correlations in DLPFC across inflammation subgroups. **(A)** TSPO mRNA was elevated in high inflammation group (*p* < 0.0001) compared to low inflammation group. **(B)** GFAP mRNA was decreased in schizophrenia compared to controls (*p* = 0.05) and was elevated in high inflammation group (*p* < 0.0001) compared to low inflammation group. **(C)** CD163 mRNA was elevated in both schizophrenia group and high inflammation group compared to controls (*p* < 0.05) and low inflammation group (*p* < 0.0001), respectively. Schizophrenia high inflammation subgroup had elevated CD163 mRNA compared to control high inflammation subgroup. Error bars depict standard error of the mean. *p ≤ 0.05, ****p < 0.0001. Purple lines with * labels comparison between diagnostic groups. Black lines with * labels comparison between inflammation groups (same for all the graphs).

TSPO mRNA levels in the DLPFC did not significantly differ in people with schizophrenia compared to controls overall [main effect of diagnosis: *F*_(1,132)_ = 1.59, *p* = 0.21], but TSPO transcript was greatly elevated in the context of high inflammation compared to the low inflammation [main effect of inflammation: *F*_(1,132)_ = 23.62, *p* = 3.28E-06]. There was no diagnosis × inflammation interaction effect on TSPO mRNA levels [*F*_(1,132)_ = 0.067, *p* = 0.80]. There was no significant difference between SCZ-high and CON-high subgroups (*p* = 0.56) (Figure 2A). In contrast, GFAP mRNA was decreased in schizophrenia compared to controls overall [main effect of diagnosis: *F*_(1,127)_ = 4.61, *p* = 0.034], but elevated in high inflammation individuals compared to low inflammation individuals [main effect of inflammation: *F*_(1,127)_ = 38.47, *p* = 7.21E-09] with no significant interaction effect [*F*_(1,127)_ = 1.46, *p* = 0.23]. There was a decrease in GFAP mRNA in SCZ-high compared to CON-high subgroup (*p* = 0.05) (Figure 2B).

We found mRNA levels of the macrophage marker CD163 were significantly elevated in schizophrenia cases compared to control cases [main effect of diagnosis: *F*_(1,127)_ = 4.00, *p* = 0.048], and were also greatly elevated in individuals with high inflammation compared to those with low inflammation [main effect of inflammation: *F*_(1,127)_ = 24.08, *p* = 2.78E-06] (Figure 2C). There was a trend level of diagnosis × inflammation interaction effect [*F*_(1,127)_ = 3.48, *p* = 0.064] on CD163 expression. Importantly, CD163 mRNA was significantly higher in the SCZ-high subgroup than the CON-high subgroup (*p* = 0.027) (Figure 2C).

### Common Microglial Marker TSPO mRNA Was More Positively Related to Astrocytic Marker GFAP mRNA Than to Microglia Marker IBA1 mRNA

**FIGURE 3:**
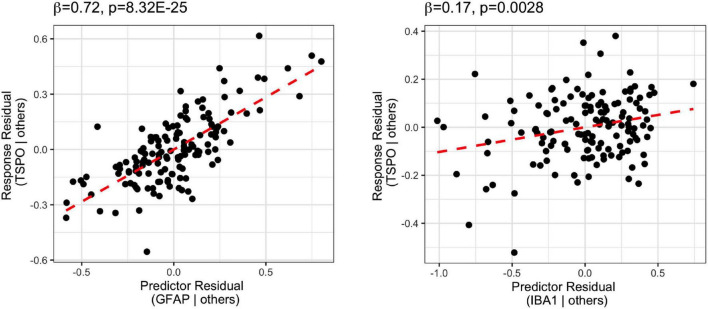
Stepwise regression analysis of TSPO mRNA vs. microglia (IBA1), astrocyte (GFAP), and macrophage (CD163) marker mRNA. The predictors of the final model were GFAP and IBA1, with GFAP most positively related to TSPO (β = 0.72, *p* = 8.32E-25).

Stepwise multiple linear regression analysis on TSPO mRNA and microglia, astrocyte, and macrophage markers (IBA1, GFAP, CD163 mRNAs; Figure 3 and Supplementary Table 3) showed that the best predictors of TSPO in the final model were GFAP mRNA and IBA1 mRNA, which accounted for 55% of the variance (adjusted *R*^2^ = 0.55) in TSPO gene expression. The degree to which GFAP mRNA levels predicted TSPO mRNA levels (β = 0.72, *p* = 8.32E-25) was more than 4-fold higher than the degree to which IBA1 mRNA levels predicted TSPO mRNA levels (β = 0.17, *p* = 0.0028). CD163 mRNA was excluded from the model due to relatively low contribution to the variance (β = 0.051, *p* = 0.44).

### CD-163 mRNA Levels in the Schizophrenia Brain Are Associated With Pro-inflammatory Macrophage Markers

**FIGURE 4:**
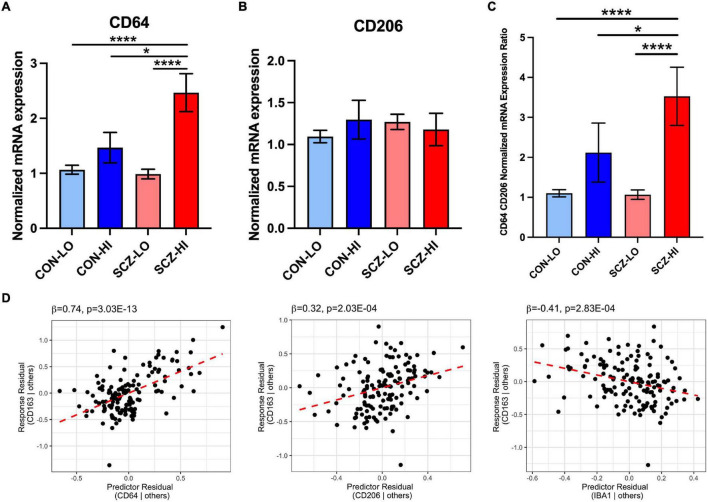
Comparison of M1 (CD64) and M2 (CD206) macrophage marker mRNA expression across inflammation subgroups. **(A)** CD64 mRNA expression was elevated in high inflammation group compared to low inflammation group [*F*_(1,130)_ = 14.916, *p* = 1.85E-04]. There was an interaction effect of diagnosis and inflammation [*F*_(1,130)_ = 4.268, *p* = 0.041]. CD64 mRNA was elevated in schizophrenia high inflammation subgroup compared to control and schizophrenia low inflammation subgroups (*p* < 0.0001) and control high inflammation subgroup (*p* < 0.05). **(B)** CD206 mRNA expression was not significantly changed across inflammation subgroups. **(C)** The ratio of CD64 and CD206 expression was significantly increased in high inflammation group compared to low inflammation group [*F*_(1,131)_ = 17.87, *p* = 4.4E-05]. There was an interaction effect of diagnosis and inflammation [*F*_(1,131)_ = 4.267, *p* = 0.041]. The CD64/CD206 ratio was significantly elevated in schizophrenia high inflammation subgroup compared to control and schizophrenia low inflammation subgroups (*p* < 0.0001) and control high inflammation subgroup (*p* < 0.05). **(D)** Regression analysis of CD163 vs. CD64, CD206, and IBA1 mRNA. Macrophage marker CD163 mRNA was positively related to CD64 and CD206 mRNA, while negatively related to IBA1 mRNA. Error bars depict standard error of the mean. *p < 0.05, **** *p* < 0.0001.

We found no significant differences in M1 macrophage marker CD64 [*F*_(1,130)_ = 2.50, *p* = 0.12] or M2 macrophage marker CD206 [*F*_(1,129)_ = 0.12, *p* = 0.73] gene expression by diagnosis. However, CD64 mRNA was elevated in the high inflammation individuals compared to the low inflammation individuals overall [main effect of inflammation: *F*_(1,130)_ = 14.82, *p* < 0.001], while CD206 expression was not significantly different based on inflammatory status [*F*_(1,129)_ = 0.46, *p* = 0.18]. We detected a significant diagnosis × inflammation interaction effect on CD64 mRNA [*F*_(1,130)_ = 4.27, *p* = 0.041] but not CD206 mRNA [*F*_(1,129)_ = 1.83, *p* = 0.18] (Figures 4A,B). CD64 mRNA was significantly increased in the SCZ-high subgroup compared to the CON-low and SCZ-low subgroups (*p* < 0.0001), and also compared to the CON-high subgroup (*p* = 0.036) (Figure 4A). CD206 did not differ between CON-high and SCZ-high subgroups (*p* = 0.73). The ratio of CD64/CD206 mRNA did not differ by diagnosis [*F*_(1,131)_ = 2.12, *p* = 0.15], but the ratio was significantly higher in high inflammation individuals compared to the low inflammation individuals overall [main effect of inflammation: *F*_(1,131)_ = 17.87, *p* = 4.4E-05] (Figure 4C). There is a significant interaction effect of diagnosis × inflammation on the ratio of CD64/CD206 mRNA [*F*_(1,131)_ = 4.27, *p* = 0.041] that mimicked the analysis of CD64 alone. The ratio of CD64/CD206 mRNA was significantly increased in the SCZ-high subgroup compared to the CON-low and SCZ-low subgroups (*p* < 0.0001), and also compared to the CON-high subgroup (*p* = 0.047). To assess the potential phenotype of CD163+ macrophages, we performed regression analysis on CD163 mRNA vs. the putative macrophage markers, CD64, CD206, and microglia marker IBA1 mRNAs. We found that all three markers contributed to the final model (adjusted *R*^2^ = 0.40), where CD64 mRNA (β = 0.74, *p* = 3.03E-13) and CD206 mRNA (β = 0.32, *p* = 0.00020) were positively related to CD163 mRNA, while IBA1 mRNA (β = −0.41, *p* = 0.00028) was negatively related to CD163 (Supplementary Table 3 and Figure 4D).

### Anti-inflammatory Macrophage Markers Are Either Decreased or Unchanged in Schizophrenia

**FIGURE 5:**
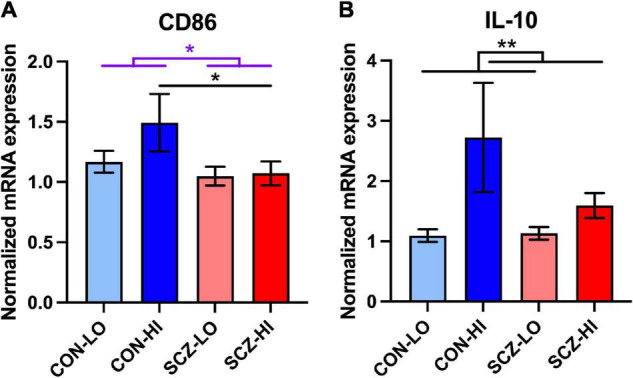
Comparison of M2b macrophage related mRNA expression in inflammation subgroups. **(A)** M2b macrophage surface marker CD86 expression was decreased in schizophrenia compared to the controls [*F*_(1,134)_ = 4,791, *p* = 0.030]. CD86 mRNA expression was lower in schizophrenia high inflammation subgroup compared to control high inflammation subgroup (*p* < 0.05). **(B)** Anti-inflammatory cytokine IL-10 expression was elevated in high inflammation group compared to low inflammation group (*p* < 0.01). Error bars depict standard error of the mean. *p < 0.05, ** *p* < 0.01.

In terms of the M2b macrophage surface marker mRNA, CD86, we found decreased expression in schizophrenia compared to controls overall [main effect of diagnosis: *F*_(1,134)_ = 4.79, *p* = 0.030]. There were no effect of inflammation [main effect of inflammation: *F*_(1,134)_ = 1.98, *p* = 0.16] or diagnosis × inflammation interaction [*F*_(1,134)_ = 1.49, *p* = 0.22] on CD86 mRNA levels. In comparing the high inflammation subgroups directly, CD86 mRNA was significantly lower in schizophrenia than controls (*p* = 0.048) (Figure 5A). For the anti-inflammatory cytokine, IL-10 gene expression did not differ between diagnostic groups [main effect of diagnosis: *F*_(1,124)_ = 0.47, *p* = 0.50]. However, IL-10, mRNA expression was elevated in high inflammation individuals compared to low inflammation individuals overall [main effect of inflammation: *F*_(1,124)_ = 9.34, *p* = 0.0027] with no diagnosis × inflammation interaction effect [*F*_(1,124)_ = 2.45, *p* = 0.12] (Figure 5B). IL-10 mRNA levels were did not differ between CON-high and SCZ-high subgroups (*p* > 0.19) (Figure 5B).

### Increased Macrophage Attractant CCL2 mRNA in Schizophrenia Is Associated With CD163 mRNA

**FIGURE 6:**
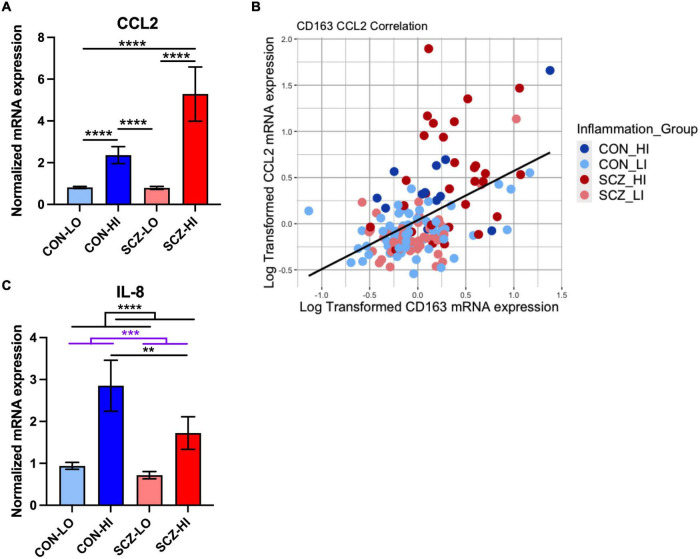
Macrophage-related chemokine gene expression in DLPFC comparison across inflammation subgroups. **(A)** Macrophage chemokine CCL2 mRNA was elevated in schizophrenia compared to controls and in high inflammation groups compared to low inflammation groups (both *p* < 0.0001). There was a significant diagnosis × inflammation interaction effect on CCL2 expression [*F*_(1,130)_ = 17.37, *p* = 5.57E-05]. **(B)** Correlations between CD163 and CCL2 mRNA expression (log transformed data). **(C)** IL-8 mRNA was decreased in schizophrenia compared to controls (*p* < 0.001) and was elevated in high inflammation group compared to low inflammation group (*p* < 0.0001). IL-8 mRNA was decreased in schizophrenia high inflammation subgroup compared to control high inflammation subgroup (*p* < 0.01). **p < 0.01, ***p < 0.001, ****p < 0.0001.

We found that mRNA levels of macrophage chemoattractant, CCL2, were elevated in schizophrenia compared to controls [main effect of diagnosis: *F*_(1,130)_ = 16.71, *p* = 7.58E-05], and were increased in high inflammation individuals compared to low inflammation individuals [main effect of inflammation: *F*_(1,130)_ = 32.08, *p* = 9.05E-08]. There was a significant diagnosis × inflammation interaction effect on CCL2 expression [*F*_(1,130)_ = 17.37, *p* = 5.57E-05], where CCL2 mRNA was more significantly increased in SCZ-high vs. SCZ-low (*p* = 1.16E-07) than in CON-high vs. CON-low (*p* = 9.38E-06) (Figure 6A). CCL2 mRNA was also significantly elevated in SCZ-high compared to CON-low (*p* = 6.84E-07) and in CON-high compared to SCZ-low subgroup (*p* = 9.04E-07), but did not differ between CON-high and SCZ-high subgroups (*p* = 0.68) or between CON-low and SCZ-low subgroup (*p* = 0.48). As predicted, we found CCL2 mRNA and CD163 mRNA levels were moderately positively correlated in the whole cohort (*r* = 0.455, *p* = 6.27E-08) (Figure 6B). Meanwhile, we also found a moderate positive correlation between CCL2 and GFAP mRNA expression (*r* = 0.54, *p* = 5.35E-12).

Contrary to our expectations, we found that mRNA levels of the chemokine secreted by macrophages, IL-8, were decreased in schizophrenia compared to controls overall [main effect of diagnosis: *F*_(1,127)_ = 14.28, *p* = 2.41E-04], but that IL-8 mRNA was significantly elevated in high inflammation individuals compared to low inflammation individuals overall [main effect of inflammation: *F*_(1,127)_ = 30.89, *p* = 1.54E-07] with no diagnosis × inflammation interaction effect [*F*_(1,127)_ = 2.40, *p* = 0.12]. IL-8 mRNA was significantly decreased in SCZ-high compared to CON-high subgroups (*p* = 0.0024) (Figure 6C). IL-8 mRNA and CD163 mRNA were moderately positively correlated overall (*r* = 0.34, *p* = 3.96E-05). However, we found these two genes were more positively correlated in CON-high (*r* = 0.72, *p* = 0.0086) and were not correlated in the SCZ-high subgroup (*r* = −0.0014, *p* = 0.99) (*Z* = 2.35, *p* = 0.018).

### Chemokine CCL2 mRNA Was Positively Correlated With Pan and Pro-inflammatory Macrophage Markers and Negatively Correlated With Anti-inflammatory Macrophage Markers in the DLPFC

**FIGURE 7:**
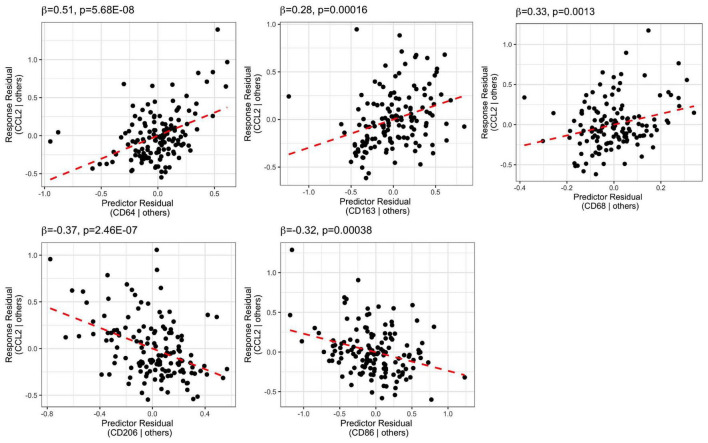
Stepwise regression analysis model of CCL2 vs. macrophage markers (CD64, CD163, CD68, CD206, CD86). CCL2 mRNA was positively related to CD64, CD163, and CD68, whereas it was negatively related to CD206 and CD86.

We performed stepwise multiple linear regression analysis for the macrophage chemoattractant, CCL2 mRNA, and the macrophages markers including CD68, CD163, CD64, CD206, and CD86 mRNAs (Supplementary Table 3 and Figure 7). The final model predictors included all the macrophage markers and accounted for 56% of CCL2 mRNA variance (adjusted *R*^2^ = 0.56). Macrophage markers CD64 mRNA (β = 0.51, *p* = 5.68E-08), CD163 mRNA (β = 0.28, *p* = 0.00016), and CD68 mRNA (β = 0.33, *p* = 0.0013) were positively related to CCL2 mRNA, while CD206 mRNA (β = −0.37, *p* = 2.46E-07) and CD86 mRNA (β = −0.32, *p* = 0.00038) were negatively related to CCL2 mRNA.

### Relationships of Transcripts of Interest and/or Inflammatory Status to Clinical Variables

Brain pH was lower in patients aligning with findings of a recent meta-analysis (Hagihara et al., 2018), although the diagnostic comparison did not quite reach significance in our study. However, brain pH was significantly decreased in both CON-high and SCZ-high subgroups compared to both low inflammation subgroups. Age at onset and duration of illness were not significantly different between SCZ-high and SCZ-low subgroups. The mean daily dose of chlorpromazine (CPZ) equivalent was higher in SCZ-high than in SCZ-low subgroup and this difference reached a trend level of statistical significance (*p* = 0.057). In addition, we found that duration of illness was negatively correlated with microglia markers IBA1 mRNA (*r* = −0.39, *p* = 0.015) and CD11c mRNA (*r* = −0.29, *p* = 0.017), and with anti-inflammatory myeloid markers CD206 mRNA (*r* = −0.27, *p* = 0.024) and CD86 mRNA (*r* = −0.36, *p* = 0.002). Duration of illness was also positively correlated with the astrocyte marker GFAP mRNA (*r* = 0.31, *p* = 0.008). Age at onset was negatively correlated with CD163 mRNA (*r* = −0.27, *p* = 0.023). Lifetime CPZ equivalents which were related to duration of illness were negatively correlated with CD11c mRNA (*r* = −0.32, *p* = 0.007), CD206 mRNA (*r* = −0.33, *p* = 0.005) and CD86 mRNA (*r* = −0.30, *p* = 0.010), but positively correlated with GFAP mRNA (*r* = 0.26, *p* = 0.028) and CCL2 mRNA (*r* = 0.40, *p* = 6E-04). Daily CPZ equivalents (average daily dose) were significantly positively correlated with CCL2 mRNA (*r* = 0.34, *p* = 0.043).

### PsychENCODE Consortium RNAseq Analysis Confirmed Many Inflammation-Related Changes and Showed Diagnostic Specific Changes in Schizophrenia

In PsychENCODE Consortium data (Table 3), we found that one inflammation related transcript, SERPINA3, was elevated in all three psychiatric disorders, schizophrenia (log2FC = 0.93, FDR = 1.62E-10), autism spectrum disorder (ASD) (log2FC = 1.15, FDR = 0.059) and bipolar disorder (BD) (log2FC = 0.53, FDR = 0.044) compared to controls. Although this SERPINA3 mRNA increase in ASD (FDR = 0.059) did not reach statistical significance and was near the level of statistical significance in BD (FDR = 0.044) compared to controls. In support of the inflammatory changes being stronger in schizophrenia, we found that IL-6 mRNA (log2FC = 0.47, FDR = 0.0021) was elevated in schizophrenia compared to controls, but was not significantly changed in either Autism or Bipolar Disorder. We identified a reduction in the microglial marker IBA1 (log2FC = −0.19, FDR = 0.014) and confirmed our reduction of CD11c mRNA (log2FC = −0.30, FDR = 2.03E-08) in schizophrenia compared to the controls, but again these changes were not identified in BD or in ASD (FDR > 0.05). Reactive astrocyte marker GFAP mRNA (log2FC = 0.14, FDR = 0.0016) was elevated in schizophrenia and Autism groups compared to control. Whereas, the macrophage marker CD163 mRNAs (log2FC = 0.35, FDR = 5.93E-04) was increased specifically in schizophrenia. Additionally, both the M2b macrophage marker [(CD86) (log2FC = −0.17, FDR = 0.024)] and the M2 related cytokine [(IL-10) (log2FC = −0.20, FDR = 0.047)] mRNAs were decreased in schizophrenia compared to the controls, but did not differ in the other diagnostic groups. Other mRNA levels were unchanged across diagnostic groups (FDR > 0.05). Considering all transcripts tested, we found 63% [SERPINA3, IL1B, Hexb, CD11c, CD68, TSPO, CD163, CD64, CD206, CD86 (unchanged genes included)] of the results matched our results of diagnosis effect reported in this current study.

**TABLE 3:** Comparison of inflammation, microglia, macrophage, and astrocyte marker mRNA levels expression to PsychENCODE Consortium RNAseq data.

Gene ID	Gene type	Gene name	CON Mean ± SE	SCZ Mean ± SE	*P-value*	ASD log2FC	SCZ log2FC	BD log2FC	ASD *P-value*	SCZ *P-value*	BD *P-value*	ASD FDR	SCZ FDR	BD FDR
ENSG00000196136	Protein coding	SERPINA3	1.40 ± 0.23	4.34 ± 0.95	**0.01**	1.154682	0.932726	0.534707	0.004154	1.14E-13	0.001707	0.059019	**1.62E-10**	**0.043636**
ENSG00000136244	Protein coding	IL6	2.31 ± 0.44	5.30 ± 1.06	0.08	0.464269	0.465058	0.07479	0.226546	0.000138	0.659473	0.539608	**0.002082**	0.874141
ENSG00000125538	Protein coding	IL1B	1.21 ± 0.13	1.49 ± 0.20	0.41	0.749573	0.113359	0.237998	0.022091	0.261783	0.083539	0.155468	0.485372	0.350419
ENSG00000204472	Protein coding	IBA1/AIF1	1.27 ± 0.11	1.13 ± 0.08	0.31	−0.083969	−0.19176	−0.17081	0.672116	0.001694	0.038942	0.871931	**0.013903**	0.238165
ENSG00000049860	Protein coding	HEXB	1.19 ± 0.15	0.96 ± 0.07	0.67	0.039163	0.005994	−0.00842	0.246838	0.585525	0.588424	0.563571	0.76967	0.840764
ENSG00000140678	Protein coding	ITGAX/CD11c	1.12 ± 0.07	0.84 ± 0.05	**4.54E-04**	0.142535	−0.30491	−0.13705	0.335824	7.55E-11	0.033545	0.653789	**2.03E-08**	0.221272
ENSG00000129226	Protein coding	CD68	1.08 ± 0.06	1.05 ± 0.05	0.66	0.360087	−0.06516	−0.09047	0.073579	0.29583	0.2882	0.29905	0.523239	0.626372
ENSG00000100300	Protein coding	TSPO	1.01 ± 0.05	1.04 ± 0.05	0.21	0.168808	0.046	0.05867	0.03077	0.073635	0.109223	0.187817	0.214385	0.398381
ENSG00000131095	Protein coding	GFAP	1.08 ± 0.07	1.05 ± 0.06	**0.034**	0.327268	0.14173	0.064309	0.003448	9.47E-05	0.20907	**0.052738**	**0.001564**	0.544286
ENSG00000177575	Protein coding	CD163	1.18 ± 0.14	1.56 ± 0.16	**0.048**	0.488340	0.34719	0.036478	0.06909	2.76E-05	0.744658	0.289608	**0.000593**	0.912843
ENSG00000150337	Protein coding	FCGR1A/CD64	1.20 ± 0.10	1.49 ± 0.14	0.12	0.478625	0.135682	0.062625	0.03739	0.055789	0.51626	0.208711	0.177603	0.798713
ENSG00000120586	Protein coding	MRC1/CD206	1.13 ± 0.07	1.27 ± 0.10	0.73	−0.821796	−0.20504	−0.3672	0.085554	0.182241	0.08953	0.325493	0.387505	0.362222
ENSG00000114013	Protein coding	CD86	1.20 ± 0.08	1.03 ± 0.06	**0.030**	0.015427	−0.17008	−0.12964	0.933679	0.003465	0.106383	0.978595	**0.024013**	0.393482
ENSG00000136634	Protein coding	IL10	1.38 ± 0.19	1.34 ± 0.11	0.50	0.306460	−0.20326	−0.14929	0.187675	0.008695	0.178123	0.492392	**0.047408**	0.503556
ENSG00000108691	Protein coding	CCL2	1.18 ± 0.11	2.43 ± 0.48	**7.58E-05**	0.311869	0.159638	0.176239	0.329127	0.105662	0.189378	0.647036	0.273516	0.518838
ENSG00000169429	Protein coding	IL8	1.25 ± 0.13	1.13 ± 0.16	**2.41E-04**	0.216911	−0.19623	−0.19385	0.522062	0.065516	0.188607	0.788986	0.198596	0.517913

*Column 1: Gene ID, gene type, and gene name; Column 2: Summary of mRNA levels and comparison p-value between schizophrenia and controls, p-values < 0.05 were highlighted in red; Column 3–5: Summary of mRNA levels from PsychENCODE Consortium RNAseq data in autism spectrum disorder (ASD), schizophrenia (SCZ), and bipolar disorder (BPD) compared to normal controls including log2 fold change (log2FC), p-value, and false discovery rate (FDR), FDR < 0.05 was highlighted in bold.*

## Discussion

**FIGURE 8:**
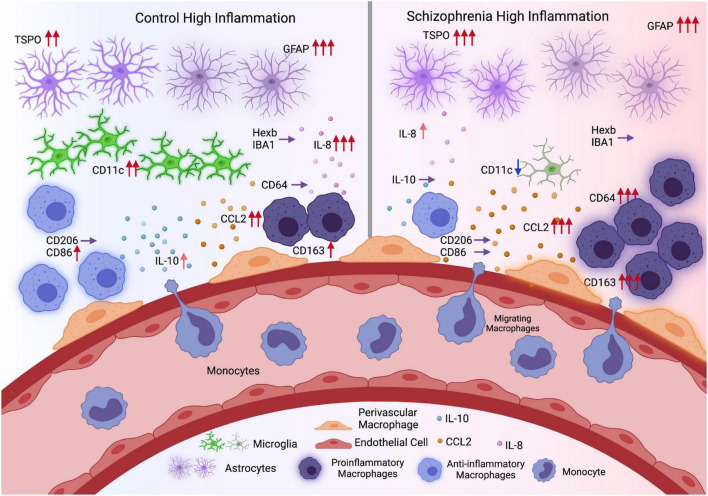
Comparison of inflammation conditions in control and schizophrenia (Hypothesis diagram). During inflammation, astrocytes may upregulate the expression of TSPO and GFAP in both controls and schizophrenia. Microglia marker IBA1 and Hexb expression remain the same in control and schizophrenia high inflammation conditions, while CD11c expression was elevated in controls but decreased in schizophrenia. Meanwhile chemokine CCL2 level was more elevated in schizophrenia during inflammation which may promote more monocyte infiltration and differentiation into pro-inflammatory macrophages (CD64). In contrast, in controls, the macrophages are more likely to maintain a balance between pro- and anti- inflammatory characteristics, with M2b macrophage marker CD86 elevated. In general, there are more pro-inflammatory signals in high inflammation schizophrenia subgroup, while in the high inflammation control subgroup, pro- and anti- inflammatory signals are more balanced (Created with BioRender.com) (Red arrows: Expression levels were elevated; more arrows indicate higher levels or more significantly elevated. Purple arrows: Expression levels were not changed. Blue arrows: Expression levels were decreased).

Inconsistencies regarding microglial activation in schizophrenia may arise due to the shared expression of several “microglia” markers across microglia, brain-resident perivascular macrophages and peripherally derived macrophages. In an attempt to disentangle the contribution of microglia vs. macrophages to neuroinflammation in schizophrenia, we measured multiple transcripts that are typically associated with microglia (Hexb, CD11c, IBA1, CD68), pro-inflammatory macrophages (CD64) anti-inflammatory macrophages (CD206, M2b: CD86, IL-10), the macrophage scavenger receptor CD163, and immune cell chemokines (CCL2, IL-8) (Figure 8). Clear differences in the direction of change in transcripts between high and low inflammation schizophrenia and high and low inflammation control groups indicate that macrophage cell populations are contributing more to the inflammatory signal in the cortex of people with schizophrenia as compared to controls. Although we acknowledge that there is some degree of overlap in expression even with these “cell-specific” or “state-specific” markers are used (Holness and Simmons, 1993; Liu et al., 2008; Ishizuka et al., 2012). CD163 in the normal brain mainly labels border-associated macrophages (BAMs) including perivascular macrophages (Kim et al., 2006) which do not express high levels of IBA1, while microglia do (Pedragosa et al., 2018). Furthermore, cells double immune-labeled with IBA1 and CD163 have distinct morphology, density and anatomical positions as compared with microglia within the human brain (Kim et al., 2006; Swanson et al., 2020). Based on the mRNA levels of multiple markers for microglia, astrocytes and macrophages (Table 4), our data indicate the immune signal found in the cortex in schizophrenia to be more related to macrophages rather than microglia (Figure 8). Furthermore, this robust increase in CD-163 mRNA is not detected to two other related psychiatric illnesses, autism and bipolar disorder, in the large transcriptomics database from PsychENCODE.

**TABLE 4:** Microglia/Macrophage markers related references.

Gene name	Related cell type	References
AIF1/IBA1	Microglia	Imai et al., 1996
HEXB		Masuda et al., 2020
ITGAX/CD11c		Benmamar-Badel et al., 2020
CD68		Hendrickx et al., 2017
TSPO	Astrocytes	Lavisse et al., 2012
GFAP		Chiu et al., 1981
CD163	Macrophage	Kristiansen et al., 2001
FCGR1A/CD64	M1 Macrophage	Thepen et al., 2000
MRC1/CD206	M2 Macrophage	Stein et al., 1992
CD86	M2b Macrophage	Zhang et al., 2015
IL10		

Overall, we found support for lower-than-normal levels of microglial transcripts in the prefrontal cortex of patients with schizophrenia (no increase in IBA1 or Hexb mRNA, reduced CD11c mRNA), in line with putative microglial quiescence and aligning with previous reports of comparable numbers of cortical microglia in people with schizophrenia compared to controls (Bergon et al., 2015; Gandal et al., 2018a,b; Snijders et al., 2021). Lower levels of microglial markers in schizophrenia was replicated in PsychENCODE results, with no apparent change in microglia markers in either autism or bipolar disorder. We also validated that IBA-1 was largely surveying cells consistent with microglia morphology, as positive correlations between our IBA1 mRNA levels and the density of anatomically defined microglia from SMRI database were detected [Array Collection Clare Beasley IBA1+ cells in the frontal cortex (see text footnote 1)]. Interestingly, CD11c is expressed in both resting microglia and activated microglia and is typically up-regulated during neuroinflammation (Wlodarczyk et al., 2014, 2015), yet we find decreased CD11c in the cortex of the high inflammation schizophrenia subgroup relative to inflamed and non-inflamed controls. CD11c+ microglia are more numerous in aging and neurodegenerative disease and are a beneficial adaptation to apoptotic and necrotic neurons *via* their increased propensity for phagocytic clearance of dying cells (Wlodarczyk et al., 2015, 2018; Benmamar-Badel et al., 2020) and amyloid plaques (Kamphuis et al., 2016). Since neither our diagnostic groups nor inflammatory subgroups differed in age, the reduction in CD11c mRNA that was specific to high inflammation schizophrenia could reflect a lack of “normal” microglial processes required to maintain brain homeostasis in the face of elevated cytokines. Combined with our findings of unchanged Hexb, CD68, and IBA1 mRNAs in high inflammation schizophrenia, as well as previous studies reporting no change in microglial cell number or density in cortical gray matter in schizophrenia (Arnold et al., 1998; Steiner et al., 2006, 2008), it seems likely that patients may not have a deficit in microglia numbers but a deficit in microglial responsiveness. Previous studies have found decreased expression of microglia-related mRNAs (Gandal et al., 2018b; Snijders et al., 2021) and decreased TSPO binding *via* PET (Collste et al., 2017; Notter et al., 2018) in schizophrenia compared to controls. This apparent microglial suppression appears abnormal in people with schizophrenia who also have evidence of active cortical inflammation.

Given that microglia can become “exhausted” in response to chronic inflammatory activation (Block and Hong, 2005; Cherry et al., 2014; Bachiller et al., 2018), we speculate that there may be heightened microglial reactivity early in the disease which then transitions into suppression of microglial responsiveness after several decades (van Berckel et al., 2008; Doorduin et al., 2009; Bloomfield et al., 2016; Di Biase et al., 2017; Hafizi et al., 2018; Notter et al., 2018). The possibility of “microglia exhaustion” is supported by electron microscopy findings demonstrating increased markers of cellular senescence within microglia in chronically ill patients compared to age-matched controls, which were also positively correlated with duration of illness in patients (Uranova et al., 2021). It is possible that senescence of microglia is related to the duration of illness in schizophrenia or to prolonged antipsychotic treatment, which may promote astrocytic reactivity.

In contrast to the patterns of expression observed for the microglial transcripts, Hexb, CD11c, IBA1, and CD68, we found that mRNAs expressed by microglia and/or astrocytes, TSPO, and GFAP, were elevated in high inflammation schizophrenia compared to low inflammation schizophrenia, suggesting astrocytes may be more actively involved in the inflammation process in schizophrenia. However, the decreased GFAP mRNA in schizophrenia compared to controls overall could be due to the greater elevation of GFAP mRNA in high inflammation controls in our study, which aligns with previous studies of GFAP expression showing no change or even decreases in schizophrenia overall (Trepanier et al., 2016). Meanwhile, we did find a strong positive relationship between TSPO mRNA and GFAP mRNA. Since TSPO expression can also be expressed by astrocytes (Lavisse et al., 2012; Notter et al., 2020), and astrocytic TSPO levels increase before microglial TSPO in Alzheimer’s disease (Tournier et al., 2020), it is plausible that astrocytes are the primary source of cortical TSPO in people with schizophrenia and elevated inflammation.

There were two macrophage marker changes that demonstrated a clear elevation in high inflammation schizophrenia compared to high inflammation controls, and one was the macrophage scavenger receptor CD163 mRNA and the other was M1 macrophage marker FcγR1/CD64. These macrophage transcript were robustly increased in the cortex of the high inflammation schizophrenia subgroup relative to all other diagnostic/inflammatory subgroups. This indicates that the cells expressing Hexb, CD11c, IBA1, and CD68 mRNAs are likely distinct from those expressing CD163/CD64 mRNA (Kim et al., 2006; Borda et al., 2008). CD163 is expressed in BAMs in normal conditions, and during inflammation CD163+ BAMs increase vascular permeability to promote leukocyte infiltration in ischemic stroke (Pedragosa et al., 2018). There is also evidence for accumulation of CD163+ macrophages in multiple sclerosis brains (Zhang et al., 2011) and in the lesions of traumatic brain injury (Zhang et al., 2012). Since both perivascular and peripheral macrophages can express CD163 (Borda et al., 2008; Saylor et al., 2018), the increased levels of CD163 mRNA reported here and previously (Cai et al., 2018; Purves-Tyson et al., 2020; Weissleder et al., 2021) in high inflammation patients could reflect an increase in either or both cell populations. CD64 usually upregulates in pro-inflammatory environment and has been a target in the treatment of chronic inflammatory diseases driven by pro-inflammatory macrophages (Akinrinmade et al., 2017). Peripheral macrophages only enter the brain under neuroinflammatory conditions (Zhang et al., 2011, 2012), so it is important to more definitively determine the origin of the increased CD163/CD64+ macrophages in schizophrenia and to establish the phenotype of these cells in order to understand their potential consequences for brain homeostasis in patients. Although we found CD163 correlated with both M1 and M2 macrophage marker, CD163 expression is not restricted to M2 macrophages (Barros et al., 2013) but can also be expressed in “M1-like” (Mori et al., 2015). Given that patients with increased CD163+ cells also have elevated pro-inflammatory cytokine mRNAs in the prefrontal cortex, it is probable that the phenotype of CD163+ macrophages found in this study is “M1-like.”

Another transcript CCL2 mRNA, which relates to macrophage recruitment, demonstrated a clear interaction effect. Like the macrophage marker, CD163/CD64, this macrophage chemoattractant was significantly increased in the high inflammation schizophrenia subgroup relative to all other subgroups. This finding provides support for the interpretation that chemotaxis of blood-borne monocytes to the brain is increased in high inflammation schizophrenia and that this is what distinguishes it from high inflammation control brain. In fact, the increase in CCL2 mRNA in these patients was the highest change of any transcript measured in any subgroup. While we do not know if the actual number of inflammatory monocytes in brain (which express high levels of CCR2) in high inflammation schizophrenia is similar to other neuroinflammatory diseases including multiple sclerosis, ischemic brain injury, and traumatic brain injury (Arakelyan et al., 2005; Semple et al., 2010), this is an important question for future comparative research. We found positive relationships between CCL2 and CD163 transcripts and the pro-inflammatory macrophage marker CD64 mRNA, but not between CCL2 transcripts and the anti-inflammatory macrophage markers CD206 mRNA and CD86 mRNA, which could indicate that these putatively “recruited” macrophages are more likely to be pro-inflammatory.(Fillman et al., 2013, 2014). CCL2, coming from either myeloid cells or astrocytes (Kim et al., 2014; Moreno et al., 2014), can influence the differentiation of tissue macrophages and promotes the production of proinflammatory cytokines including IL-6, IL-1β, and TNF-α (Yang et al., 2011; Gschwandtner et al., 2019) which are all elevated in the brains of people with high inflammation schizophrenia (Fillman et al., 2013, 2014). Reciprocally, pro-inflammatory cytokines induce the expression of CCL2 (Huang et al., 2000; Thibeault et al., 2001; Harkness et al., 2003). Therefore, elevated pro-inflammatory cytokines may promote CCL2 expression, macrophage transmigration, and macrophage differentiation into a more pro-inflammatory phenotype and may propagate a feed-forward cycle of inflammation in the prefrontal cortex or people with schizophrenia.

We found that CD163 mRNA was negatively related to microglia marker IBA1 mRNA, consistent with suppression of microglia with activation and/or infiltration of brain macrophages (Greenhalgh et al., 2018). Indeed, infiltrating macrophages can suppress microglia mediated inflammation and phagocytosis after CNS injury (Greenhalgh et al., 2018). However, healthy microglia, can limit macrophage infiltration after acute demyelination (Plemel et al., 2020) and prevent immune cell invasion in an AD mouse model (Unger et al., 2018), highlighting the complex interplay between local (brain-resident) and peripheral immune cells to achieve both an appropriate level of neuroinflammation and the resolution of neuroinflammation. Phagocytosis and chemotaxis are compromised in aged microglia, which may lead to a more pro-inflammatory microenvironment (Rawji et al., 2016). If the normal immunoregulatory roles of microglia are impaired then this could lead to an excess of peripheral immune cells permitted to enter the brain. Altering the brain microenvironment from pro-inflammatory to anti-inflammatory may therefore rejuvenate resident immune cells to maintain healthy brain function in people with schizophrenia and neuroinflammation. We also found a strong positive correlation between anti-inflammatory macrophage markers (CD206 mRNA, CD86 mRNA) and the microglia marker IBA1 mRNA, again suggesting that microglia may be benefit from anti-inflammatory macrophage-derived factors. Our results support findings that show a pro-inflammatory environment suppresses microglia, while an anti-inflammatory environment may support microglia homeostasis (Bachiller et al., 2018; Chagas et al., 2020).

### Limitations of the Study

While we have found several very significant changes in glial and immune cell related transcripts in the cortex of people with schizophrenia which often show a greater effect size in high inflammation schizophrenia compared to both low inflammation schizophrenia and controls, we also acknowledge that our study has several limitations. Given the large cohort and the logistical limitations of postmortem research we did not anatomically map transcriptional changes in cortical cells using in-situ hybridization or snRNA seq. Thus, we do not know the source of the mRNA changes, nor do we know the extent to which macrophage density or origin is changed (Bennett et al., 2018). Furthermore, we did not measure the protein levels of the transcripts in our study and future studies would be needed to compare the protein levels of microglia-, macrophage-, and astrocyte-associated proteins across inflammatory subgroups in schizophrenia and controls to bolster our current finding. Additionally, we did not stratify the PsychENCODE RNA seq data by low and high inflammation subgroups and we may find changes in either ASD or BD when analyzing by inflammation subgroups.

We were also not able to determine the direct effects of long-term antipsychotics on our transcripts of interest in mammalian brain, which is an important avenue for future research given that some of the microglia, macrophage and chemokine (CD11c, CD86, CD206, and CCL2) mRNA measured correlated with daily antipsychotic dose in the current study. However, daily CPZ equivalents were not well-matched between low and high inflammation schizophrenia subgroups (higher daily dose in high vs. low inflammation schizophrenia subgroups at a trend level). Thus, we cannot rule out that some of the inflammatory changes we detected are the result of antipsychotics; it is also possible that a more inflamed brain necessitates a higher dose of antipsychotic treatment. In support that inflammatory changes are not simply the direct result of treatment, antipsychotics are known to lower the peripheral levels of cytokines (Miller et al., 2011) and decrease cytokines in the rodent brain (Sugino et al., 2009; Macdowell et al., 2013; Purves-Tyson et al., 2019).

## Conclusion

Here we propose that macrophages, rather than microglia, may play a more salient role in neuroinflammation in schizophrenia. We showed that various macrophage markers – but not microglial markers–are increased in the post-mortem prefrontal cortex of patients, especially in a subset of people with schizophrenia that also have high inflammation as previously described by us. Our results are consistent with these macrophages being more likely to be pro-inflammatory in nature, which may disrupt immune homeostasis in the brain. Moreover, we found that anti-inflammatory macrophage markers are positively related to microglia markers, suggesting people with schizophrenia may be lacking a normal macrophage-microglia relationship. Our work provides an alternative framework to understand neuroinflammation in schizophrenia. In future, the relative contributions of different cell types to neuroinflammatory signaling using single-nuclei RNA-seq could be used to confirm these possibilities and to anatomically map changes in pro- and anti-inflammatory transcripts to brain cells. This may help us to determine how to restore the imbalance in cellular immune responses in the brains of people with schizophrenia.

## Data Availability Statement

The original contributions presented in this study are included in the article/Supplementary Material, further inquiries can be directed to the corresponding author.

## Ethics Statement

The studies involving human participants were reviewed and approved by Human Research Ethics Committee at University of NSW (#HREC: HC12435; HC17826). Written informed consent for participation was not required for this study in accordance with the national legislation and the institutional requirements.

## Author Contributions

YZ, MW, and CW: study design. YZ, MW, CM, CL, RD, and CW: methodology. YZ, CM, RD, and CW: data analysis, validation, and writing—original draft. CW: funding acquisition and supervision. MW and CW: resources. All authors contributed to the writing—review and editing and approved the submitted version.

## Conflict of Interest

The authors declare that the research was conducted in the absence of any commercial or financial relationships that could be construed as a potential conflict of interest.

## Publisher’s Note

All claims expressed in this article are solely those of the authors and do not necessarily represent those of their affiliated organizations, or those of the publisher, the editors and the reviewers. Any product that may be evaluated in this article, or claim that may be made by its manufacturer, is not guaranteed or endorsed by the publisher.

## Footnotes

1
http://sncid.stanleyresearch.org/
