# Supplementary material for: Distinct Phenotypes of Inflammation Associated Macrophages and Microglia in the Prefrontal Cortex Schizophrenia Compared to Controls
Source: Front Neurosci. 2022 Jun 30;16:858989. doi: 10.3389/fnins.2022.858989 (PMC9279891; doi:10.3389/fnins.2022.858989)
Supplement: Supplementary file 1 [file Data_Sheet_1.docx]

Supplementary Table 1: Stepwise multiple linear regression model information

| Model Dependent Variable | Model Predictors | Unstandardized $\boldsymbol{\beta}$ | Standardized $\boldsymbol{\beta}$ | t | p value |
| --- | --- | --- | --- | --- | --- |
| CD163 | CD64 | 0.822 | 0.743 | 8.079 | 3.03E-13 |
|  | CD206 | 0.448 | 0.323 | 3.818 | 2.03E-04 |
|  | IBA1 | -0.509 | -0.408 | -3.726 | 2.83E-04 |
| TSPO | GFAP | 0.567 | 0.718 | 12.639 | 8.32E-25 |
|  | IBA1 | 0.103 | 0.173 | 3.048 | 0.0028 |
| CCL2 | CD64 | 0.607 | 0.513 | 5.750 | 5.68E-08 |
|  | CD206 | -0.554 | -0.373 | -5.437 | 2.46E-07 |
|  | CD163 | 0.296 | 0.277 | 3.893 | 0.00016 |
|  | CD86 | -0.183 | -0.322 | -3.643 | 0.00038 |
|  | CD68 | 0.675 | 0.333 | 3.284 | 0.0013 |

Supplementary Table 2: The correlation of gene mRNA expression and duration of illness, lifetime CPZ equivalents, and daily CPZ equivalents. * labels p value < 0.05

| **Gene** | **Age at onset** | | **Duration of illness** | | **Lifetime CPZ equivalents** | | **Daily CPZ equivalents** | |
| --- | --- | --- | --- | --- | --- | --- | --- | --- |
|  | Spearman’s $\rho$ | P value | Pearson’s r | P value | Spearman’s $\rho$ | P value | Spearman’s $\rho$ | P value |
| IBA1 | -0.031 | 0.793 | -0.296 | 0.015* | -0.168 | 0.174 | -0.045 | 0.716 |
| Hexb | 0.075 | 0.529 | -0.027 | 0.829 | -0.006 | 0.964 | 0.034 | 0.782 |
| CD11c | 0.038 | 0.751 | -0.288 | 0.017* | -0.322 | 0.007* | -0.213 | 0.081 |
| CD68 | -0.086 | 0.474 | -0.156 | 0.197 | -0.127 | 0.303 | -0.065 | 0.601 |
| TSPO | -0.157 | 0.188 | 0.121 | 0.314 | 0.118 | 0.327 | 0.067 | 0.579 |
| GFAP | -0.077 | 0.522 | 0.313 | 0.008* | 0.263 | 0.028* | 0.088 | 0.466 |
| CD163 | -0.269 | 0.023* | 0.103 | 0.404 | 0.134 | 0.276 | 0.145 | 0.237 |
| CCL2 | -0.176 | 0.139 | 0.067 | 0.584 | 0.400 | 6E-04* | 0.243 | 0.043* |
| IL8 | -0.094 | 0.432 | -0.048 | 0.703 | 0.059 | 0.635 | 0.130 | 0.294 |
| CD64 | -0.152 | 0.204 | 0.086 | 0.480 | 0.192 | 0.115 | 0.152 | 0.216 |
| CD206 | 0.158 | 0.185 | -0.271 | 0.024* | -0.334 | 0.005* | -0.216 | 0.074 |
| CD86 | 0.064 | 0.592 | -0.364 | 0.002* | -0.304 | 0.010* | -0.149 | 0.218 |
| IL10 | -0.119 | 0.336 | 0.034 | 0.789 | -0.099 | 0.434 | -0.186 | 0.138 |

Supplementary Table 3: Number of outliers removed from the analysis of all the qPCR target genes (in each inflammatory subgroups)

| Gene Name | Control Low (n=57) | Control High (n=12) | Schizophrenia Low (n=42) | Schizophrenia High (n=30) |
| --- | --- | --- | --- | --- |
| AIF | 2 | 1 | 2 | 2 |
| HEXB | 1 | 1 | 2 | 2 |
| ITGAX/CD11c | 2 | 1 | 2 | 2 |
| CD68 | 4 | 1 | 2 | 2 |
| TSPO | 3 | 1 | 1 | 0 |
| GFAP | 6 | 1 | 1 | 1 |
| CD163 | 5 | 1 | 2 | 2 |
| FCGR1A/CD64 | 3 | 1 | 2 | 1 |
| MRC1/CD206 | 3 | 0 | 2 | 1 |
| CD86 | 1 | 0 | 2 | 0 |
| IL10 | 4 | 1 | 4 | 2 |

Supplementary Figure 1: Microglia-related gene expression in DLPFC across inflammation subgroups. (Scatter plot)

Supplementary Figure 2 Microglia, astrocyte and macrophage marker mRNA expression and correlations in DLPFC across inflammation subgroups.

Supplementary Figure 3:Comparison of M1 (CD64), M2 (CD206), M2b (CD86, IL-10) macrophage marker mRNA expression across inflammation subgroups.

Supplementary Figure 4 Macrophage-related chemokine gene expression in DLPFC comparison across inflammation subgroups.

Supplementary Table 4 The correlation of gene mRNA expression and BMI.

| **Gene** | **BMI** | |
| --- | --- | --- |
|  | Pearson’s r /Spearman’s rho | P value |
| IBA1 | 0.043 | 0.738 |
| Hexb | 0.021 | 0.866 |
| CD11c | 0.094 | 0.462 |
| CD68 | 0.111 | 0.384 |
| TSPO | 0.032 | 0.804 |
| GFAP | 0.082 | 0.518 |
| CD163 | 0.242 | 0.054 |
| CCL2 | 0.181 | 0.151 |
| IL8 | 0.085 | 0.506 |
| CD64 | 0.186 | 0.142 |
| CD206 | -0.003 | 0.979 |
| CD86 | -0.061 | 0.632 |
| IL10 | 0.052 | 0.687 |

Supplementary Figure 5 The comparison of life style related variables (suicide, alcohol consumption, and smoking) between diagnostic groups and among inflammation subgroups A) Percentage of individuals that committed suicide. There was significantly higher percentage of individuals who committed suicide in schizophrenia (χ^2^(1)=16.09, p=6.05E-05) and in low and high inflammation schizophrenia subgroups compared to both control groups ((χ^2^(3)=22.44, p=5.29E-05) There was no difference between low and high inflammation groups ((χ^2^(1)=0.77, p=0.38). B) Percentage of individuals who have none, low, or high amount of alcohol. There was significantly higher percentage of individuals that consume more alcohol in controls than in schizophrenia ((χ^2^(2)=11.28, p=3.55E-03) (comparison in inflammation subgroups: χ^2^(6)=17.65, p=7.17E-03). There was no difference between low and high inflammation groups ((χ^2^(1)=5.36, p=0.07).C) Percentage of individuals who smoked. There was significantly higher percentage of individuals who smoked in schizophrenia (χ^2^(1)=8.26, p=4.06E-03) and in low and high inflammatory schizophrenia subgroups compared to both control groups ((χ^2^(3)=15.15, p=1.69E-03). There was no difference between low and high inflammation groups ((χ^2^(1)=0.02, p=0.88).
